# Maintenance of a Schwann-Like Phenotype in Differentiated Adipose-Derived Stem Cells Requires the Synergistic Action of Multiple Growth Factors

**DOI:** 10.1155/2017/1479137

**Published:** 2017-07-16

**Authors:** Alice E. Mortimer, Alessandro Faroni, Mahmut A. Kilic, Adam J. Reid

**Affiliations:** ^1^Blond McIndoe Laboratories, Division of Cell Matrix Biology and Regenerative Medicine, School of Biological Sciences, Faculty of Biology, Medicine and Health, University of Manchester, Manchester Academic Health Science Centre, Manchester M13 9PL, UK; ^2^Department of Biophysics, Faculty of Medicine, Adnan Menderes University, Aydin, Turkey; ^3^Department of Plastic Surgery & Burns, University Hospitals of South Manchester, Manchester Academic Health Science Centre, Manchester, UK

## Abstract

Differentiating human adipose-derived stem cells (ASCs) towards Schwann cells produces an unstable phenotype when stimulating factors are withdrawn. Here, we set out to examine the role of glial growth factor 2 (GGF-2) in the maintenance of Schwann-like cells. Following ASC differentiation to Schwann-like cells, stimulating factors were withdrawn such that cells either remained in media supplemented with all stimulating factors, GGF-2 alone, or underwent complete withdrawal of all factors. Furthermore, each stimulating factor was also removed from the growth medium individually. At 72 hours, gene (qRT-PCR) and protein (ELISA) expression of key Schwann cell factors were quantified and cell morphology was analysed. Cells treated with GGF-2 alone reverted to a stem cell morphology and did not stimulate the production of brain-derived neurotrophic factor (BDNF), regardless of the concentration of GGF-2 in the growth medium. However, GGF-2 alone increased the expression of Krox20, the main transcription factor involved in myelination, relative to those cells treated with all stimulating factors. Cells lacking fibroblast growth factor were unable to maintain a Schwann-like morphology, and those lacking forskolin exhibited a downregulation in BDNF production. Therefore, it is likely that the synergistic action of multiple growth factors is required to maintain Schwann-like phenotype in differentiated ASCs.

## 1. Introduction

Tissue engineering strategies have sought to harness the regenerative potential of Schwann cells (SCs) to aid peripheral nerve repair [1]. Much focus has been on the use of stem cells, particularly those derived abundantly from adipose tissue [2, 3]. Adipose derived stem cells (ASCs) differentiate into Schwann-like cells (dASC) when treated with a combination of forskolin, glial growth factor 2 (GGF-2), fibroblast growth factor (FGF-2), and platelet-derived growth factor (PDGF) [2, 3]. dASC possess many SC characteristics, including a spindle-shaped morphology, a similar secretome, and the ability to induce neurite regeneration [2–4].

Whilst rat dASCs have been used successfully in rodent in vivo models, evidence is lacking regarding the efficacy of human dASCs [4]. Given that the original differentiation protocol was developed for rat bone marrow stem cells, it is unsurprising that human dASCs revert to their stem cell phenotype upon withdrawal of stimulating factors [4]. Thus, further characterisation of dASCs is required prior to clinical translation.

GGF-2 is a soluble form of neuregulin 1 (NRG1), and in SCs, NRG1 allows for the survival of SC precursors and is involved in producing myelinating cells [5, 6]. It has recently been shown that mouse ASCs lacking GGF-2 reduce their expression of SC phenotype markers [7]. Here, we examine whether GGF-2 is sufficient alone to maintain critical Schwann-like properties by examining dASCs ability to maintain a Schwann-like morphology, produce brain-derived neurotrophic factor (BDNF), and express Krox20 (the main transcription factor involved in myelination) [5].

## 2. Materials and Methods

### 2.1. Cell Harvest, Isolation, and Differentiation

Adipose tissue was harvested from female surgical patients at the University Hospital of South Manchester, UK. Patients were fully consented, and procedures were approved by the National Research Ethics Committee (NRES 13/SC/0499). A previously described protocol was used for the isolation and differentiation of ASCs [4]. Briefly, adipose tissue was first minced with a razor blade and then underwent 60 minutes of enzymatic digestion using 0.2% (*w*/*v*) collagenase (Life Technologies, Paisley, UK). A 100 *μ*m nylon mesh (Merck Millipore UK, Watford, UK) was then used to filter the dissociated tissue, and stromal vascular fraction (SVF) was obtained by centrifugation at 300*g* for 10 minutes. SVF was incubated for 1 minute with red blood cell lysis buffer (Sigma-Aldrich) to remove red blood cells, recentrifuged (10 minutes at 300*g*), and the resulting pellet of ASC was plated in 75 cm^2^ flasks in stem cell growth medium (SCGM) consisting of alpha minimum essential medium [*α*MEM (Sigma-Aldrich)] supplemented with 10% (*v*/*v*) fetal bovine serum [FBS (LabTech, Uckfield, UK)], 2 mM L-glutamine (GE Healthcare UK, Little Chalfont, UK), and 1% (*v*/*v*) penicillin–streptomycin solution. For Schwann cell differentiation, subconfluent ASC cultures were supplemented with 1 mM *β*-mercaptoethanol (Sigma-Aldrich) for 24 hours and preconditioned for 72 hours with 35 ng/mL all-trans-retinoic acid (Sigma-Aldrich). Following preconditioning, the cell culture medium was replaced with SCGM supplemented with 14 *μ*M forskolin (Sigma-Aldrich), 5.46 nM GGF-2 (a gift from Acorda Therapeutics, Ardsley, NY, USA), 10 ng/mL basic fibroblast growth factor [FGF-2 (Peprotech EC, London, UK)], and 5 ng/mL platelet-derived growth factor [PDGF (Peprotech EC, London, UK)] for 14 days. This same supplemented media was used for cell maintenance during culture and passaging until further experimentation.

### 2.2. Experimental Setup

dASCs were plated onto 12-well plates at a density of 100,000 cells per well (unless otherwise stated) and maintained in growth medium with all stimulating factors for 48 hours. Media was then replaced according to the individual experiment (see below), and cells were cultured for 72 hours, after which outcome measures described below were assessed. Experimental and technical triplicates were used throughout. Media was replaced at 48 hours with *α*MEM supplemented with only GGF-2 (dASC^−/+GGF^) at concentrations of 0.273 nM, 2.73 nM, 5.46 nM, and 10.92 nM. Withdrawal of each factor individually (dASC^+/−[forskolin, GGF, FGF, or PDGF]^) was also carried out, and in each case, a positive control of normal growth medium (dASC^+^) and negative control of withdrawal of all factors (dASC^−^) were used.

### 2.3. Morphology Analysis

Light microscopy and phalloidin staining of cells were undertaken at the experimental endpoint. Staining was carried out according to previously described methods [4]. Briefly, 5000 cells per well were plated in order to better visualize individual cell morphology. At the endpoint, cells were washed with phosphate buffer saline (PBS) solution and fixed with 4% (*w*/*v*) paraformaldehyde for 15 minutes at room temperature. Following permeabilisation with 0.2% (*v*/*v*) Triton X-100 (Sigma-Aldrich) for 20 minutes, cells were stained for 20 minutes in the dark with Alexa 488-conjugated phalloidin (1 : 40, Life Technologies, USA). After PBS washes, cells were imaged using an Olympus IX51 fluorescent microscope at 4x magnification. 15 images of cells in each experimental condition were taken, and aspect ratio (AR) for each cell (longest cell length/narrowest cell width) was determined using Image J (National Institutes of Health, USA).

### 2.4. Real-Time Polymerase Chain Reaction (qPCR)

Cells were collected in RNAprotect reagent, and RNA was extracted using an RNeasy Plus Mini Kit according to manufacturer's instructions (Qiagen). RNA was quantified using a NanoDrop ND-100 spectrophotometer (Thermo-Fisher Scientific, USA). The RT2 First Strand kit (Qiagen) was then used to reverse transcribe RNA. RT2 qPCR SYBR Green Mastermix and a Corbett Rotor Gene 6000 real-time cycler (Qiagen) were used to perform qPCR. Primer sequences are Krox20 [forward sequence: 5′-AACGGAGTGGCCGGAGAT-3′, reverse sequence: 5′-ATGGGAGATCCAACGACCTCT T-3′ (Sigma-Aldrich)], 18s [Qiagen (Cat number PPH05666E-200)] and BDNF [Qiagen (Cat number PPH00569F-200)]. All reactions were carried out in quadruplicate, and the following protocol was used: activation of HotStart DNA Taq polymerase by heating at 95°C for 10 minutes and followed by 45 cycles of 95°C for 15 seconds, 55°C for 30 seconds, and 72°C for 30 seconds. Melting curve analysis was performed in order to confirm specificity of primers. To analyse the qPCR data, the ΔΔCt method was employed, normalising data to the housekeeping gene, 18s.

### 2.5. Enzyme-Linked Immunosorbent Assay (ELISA)

ELISA kits to detect BDNF in cell culture supernatant (RayBiotech, USA) were used according to manufacturer's instructions. Plates were read at 450 nm using an Asys UVM-340 microplate spectrophotometer (Biochrom). Protein concentrations of the samples were calculated by interpolating their absorbency values on the standard curve obtained using recombinant BDNF protein.

### 2.6. Statistical Analysis

One-way analysis of variance (with Tukey test) and unpaired t tests were performed using Graphpad Prism 7.0 software (Graphpad Software Inc., USA). *P* values of <0.05 were considered statistically significant.

## 3. Results

### 3.1. dASC^−/+GGF^ Revert to a Stem Cell Morphology

**Figure 1:**
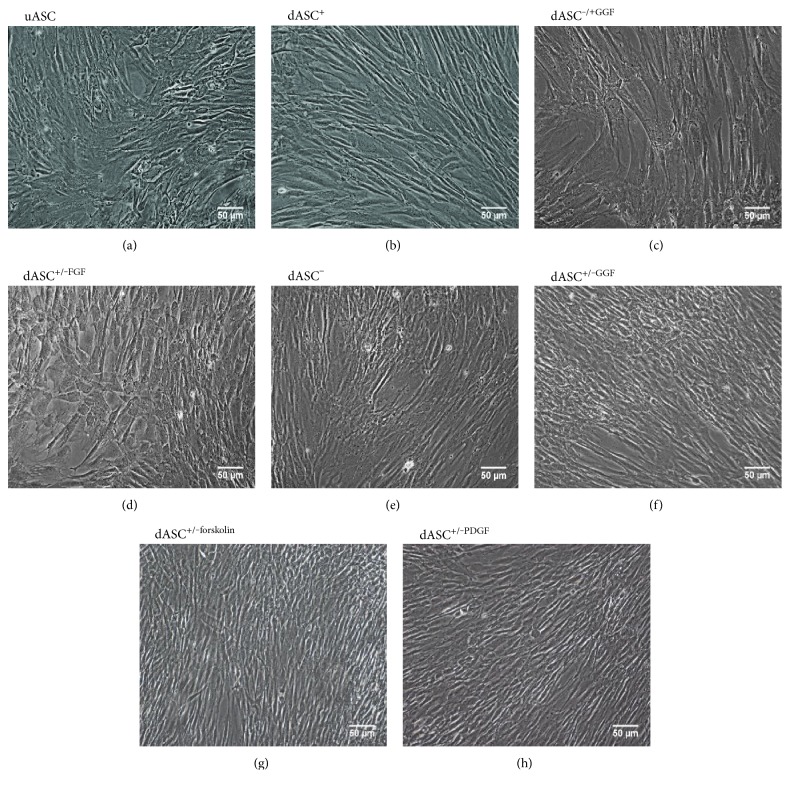
GGF-2 is not sufficient alone to maintain a spindle-shaped morphology, and FGF-2 is necessary to produce the Schwann-like morphology. (a) Undifferentiated cells display a flattened, fibroblast-like appearance. (b) dASC^+^ elongate and take on a spindle shape. (c) dASC^−/+GGF^ revert to a stem-like morphology, as do (d) dASC^+/−FGF^ and (e) dASC^−^, whilst (f) dASC^+/−GGF^, (g) dASC^+/−forskolin^, and (h) dASC^+/−PDGF^ all retain a spindle-shaped appearance. All cells were photographed at 10x magnification.

Undifferentiated ASC (uASC, Figure 1(a)) stimulated to dASC^+^ (Figure 1(b)) produced cells with an elongated, spindle-shaped morphology. dASC^*−*/+GGF^ reverted to a stem cell-like morphology, as did dASC^+/*−*FGF^ and dASC*^−^* (Figures 1(c), 1(d), and 1(e)), whilst dASC^+/*−*GGF^, dASC^+/*−*forskolin^, and dASC^+/*−*PDGF^ retained their spindle shape (Figures 1(f), 1(g), and 1(h)).

**Figure 2:**
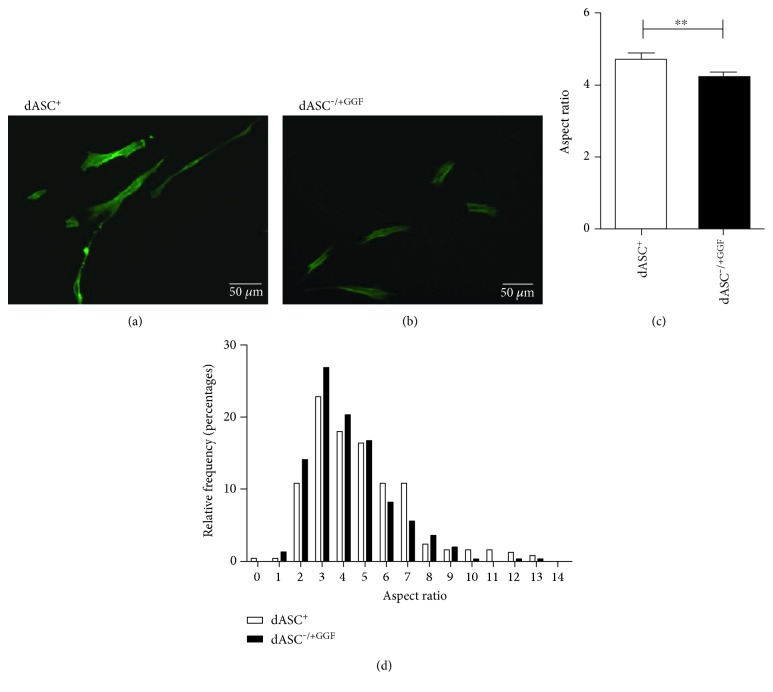
Cells treated with all stimulating factors have an increased aspect ratio compared to those treated with GGF-2 alone. (a) Spindle-shaped cells are clearly visible in dASC^+^ phalloidin-stained cells. Cells were photographed at 4x magnification. (b) dASC^−/+GGF^phalloidin-stained cells exhibit fewer spindle-shaped cells. (c) dASC^+^ had a significantly higher AR when compared to dASC^−/+GGF^ (4.752 ± 0.144 in dASC^+^ versus 4.248 ± 0.107 in dASC^−/+GGF^, ^∗∗^*P* < 0.01, *n* = 3). (d) A total of 249 dASC^+^ cells and 304 dASC^−/+GGF^ cells were analysed, and the relative frequency of each AR was determined. There was a higher percentage of cells with AR < 5 in the dASC^−/+GGF^ group (63% versus 53% in dASC^+^) and a higher percentage of spindle-shaped cells (AR ≥ 5) in the dASC^+^ group (47% versus 37% in dASC^−/+GGF^).

There were more spindle-shaped dASC^+^ (Figure 2(a)) compared to dASC^-/+GGF^ (Figure 2(b)). dASC^+^ had a significantly higher AR when compared to dASC^*−*/+GGF^ (Figure 2(c)). The relative frequencies of cells of each AR confirm that in dASC^*−*/+GGF^, there is a reduced frequency of cells with AR > 5 (Figure 2(d)).

### 3.2. dASC^−/+GGF^ Have a Phenotype Similar to dASC^−^

**Figure 3:**
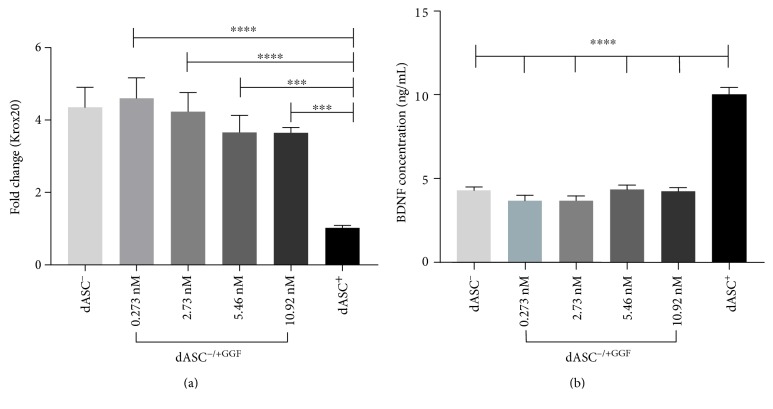
Krox20 gene expression and BDNF production in cells maintained in GGF-2 alone is similar to that of cells in which all stimulating factors have been withdrawn. (a) dASC^−^ had a 4.351 ± 0.552-fold increase in the expression of Krox20 (^∗∗∗∗^*P* < 0.0001, *n* = 3, *N* = 3); dASC^−/+GGF [0.273 nM]^ cells had a 4.599 ± 0.568-fold increase (^∗∗∗∗^*P* < 0.0001, *n* = 3, *N* = 3); dASC^−/+GGF [2.73 nM]^ cells a 4.23 ± 0.527-fold increase (^∗∗∗∗^*P* < 0.0001, *n* = 3, *N* = 3); dASC^−/+GGF [5.46 nM]^ cells a 3.657 ± 0.468-fold increase (^∗∗∗^*P* < 0.001, *n* = 3, *N* = 3); and dASC^−/+GGF [10.92 nM]^ cells a 3.648 ± 0.148 (^∗∗∗^*P* < 0.001, *n* = 3, *N* = 3) in Krox20 expression compared to dASC^+^ cells. There was no significant difference in expression between dASC^−/+GGF^ and dASC^−^. (b) dASC^+^ secrete 10 ± 0.440 ng/mL BDNF compared to 4.32 ± 0.184 ng/mL in dASC^−^, 3.673 ± 0.314 ng/mL in dASC^−/+GGF [0.273 nM]^, 3.695 ± 0.266 ng/mL in dASC^−/+GGF [2.73 nM]^, 4.351 ± 0.257 ng/mL in dASC^−/+GGF [5.46 nM]^, and 4.243 ± 0.208 ng/mL in dASC^−/+GGF [10.92 nM]^ (^∗∗∗∗^*P* < 0.0001 for all experimental groups compared to dASC^+^, *n* = 3, *N* = 3).

dASC^*−*/+GGF^ upregulated Krox20 expression compared to dASC^+^ (Figure 3(a)) but failed to maintain BDNF production levels regardless of GGF-2 concentration (Figure 3(b)). In each case, the pattern of expression was similar to that in dASC*^−^*.

### 3.3. Forskolin Is Necessary for BDNF Production

**Figure 4:**
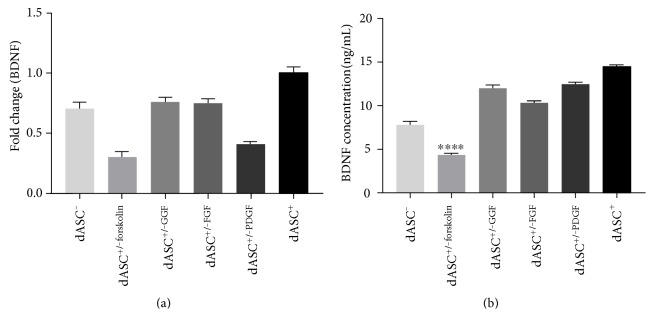
Forskolin may be involved in the production of BDNF. (a) PCR analysis of BDNF expression shows that both dASC^+/−forskolin^ and dASC^+/−PDGF^ downregulate their expression of BDNF compared to all other groups (fold change 0.705 ± 0.051 in dASC^−^, 0.302 ± 0.047 in dASC^+/−forskolin^, 0.761 ± 0.035 in dASC^+/−GGF^, 0.749 ± 0.037 in dASC^+/−FGF^, and 0.41 ± 0.017 in dASC^+/−PDGF^ compared to 1 ± 0.044 in dASC^+^. (b) ELISA protein analysis shows that dASC^+/−forskolin^ have a large downregulation of BDNF production compared to all other groups (4.354 ± 0.120 ng/mL in dASC^+/−forskolin^ versus 7.784 ± 0.413 ng/mL in dASC^−^, 12 ± 0.435 ng/mL in dASC^+/−GGF^, 10.33 ± 0.197 ng/mL in dASC^+/−FGF^, 12.46 ± 0.158 ng/mL in dASC^+/−PDGF^, and 14.48 ± 0.128 ng/mL in dASC^+^; ^∗∗∗∗^*P* < 0.0001, *n* = 3).

dASC^+/*−*forskolin^ and dASC^+/*−*PDGF^ had significantly reduced gene expression of BDNF when compared to all other groups (Figure 4(a)). dASC^+/*−*forskolin^ also had significantly reduced BDNF protein production compared to all other groups (Figure 4(b)).

## 4. Discussion

The use of ASCs in peripheral nerve injury has demonstrated real promise in experimental models [2, 3]; however, clinical translation is hindered by a rapid lack of stability in human dASCs when growth factor treatment is withdrawn [4]. Given the importance of NRG1 signalling in SC biology, we postulated that GGF-2 would be sufficient to maintain dASCs after withdrawal of other growth factors. Our results suggest that the maintenance of this phenotype is much more complex and relies on the synergistic actions of multiple growth factors. This will be crucial to understand prior to potential clinical use of this stem cell therapy for peripheral nerve injury.

We found that GGF-2 alone was unable to maintain the spindle-shaped morphology of dASCs. Interestingly, cells lacking FGF-2 also reverted to a uASC-like morphology. Stimulating rat SCs with FGF-2 in vitro induces the characteristic spindle shape, and ASCs cultured with FGF-2 maintain a spindle-shaped morphology [8, 9]. This suggests that FGF-2 may be involved in producing a Schwann-like morphology.

We found that dASC^−/+GGF^ had reduced BDNF production when compared to dASC^+^, as did dASC^+/−forskolin^. We can therefore suggest that GGF-2 is not sufficient alone to produce BDNF, but that forskolin, acting synergistically with other factors may be necessary for BDNF production. Forskolin acts through intracellular cyclic adenosine monophosphate (cAMP), and a recent study found that cAMP response element-binding protein (CREB) is instrumental in allowing for BDNF production by ASCs [10, 11]. The authors found that rat ASCs stimulated with forskolin rapidly increased their BDNF production and expression of phosphorylated CREB [11]. Hence, forskolin is likely to be involved in BDNF production [10, 11]; however, the mechanism in human ASCs remains to be elucidated.

NRG1 controls many aspects of myelination in SCs [6, 12, 13]. NRG1 signalling allows for myelination with low level stimulation but initiates demyelination with sustained activation [6, 14–16]. We investigated whether a similar effect would be seen in dASCs by stimulating cells with GGF-2 at increasing concentrations and quantifying Krox20 expression. dASC^−/+GGF^ had a significant increase in Krox20 expression compared to dASC^+^; however, there was no difference in expression between dASC^−/+GGF^ and dASC^−^ cells. This suggests that other growth factors may have an inhibitory action on Krox20 which requires further enquiry.

## 5. Conclusions

GGF-2 alone is insufficient to maintain Schwann-like properties achieved in dASCs. Cells lose their characteristic morphology and downregulate BDNF. However, we noted that cells treated with only GGF-2 increase their expression of Krox20 relative to those treated with all factors, suggesting that other factors have an inhibitory action on Krox20 expression. Forskolin appears necessary for BDNF production, and FGF-2 is likely responsible for Schwann-like morphology. Therefore, it is likely that the synergistic action of growth factors sustain the Schwann-like properties of dASC and further work in this field is required prior to clinical use of these cells.
